# Exploring the Potential of Exosomes as Biomarkers in Tuberculosis and Other Diseases

**DOI:** 10.3390/ijms25052885

**Published:** 2024-03-01

**Authors:** Rakesh Arya, Bimal Prasad Jit, Vijay Kumar, Jong Joo Kim

**Affiliations:** 1 Department of Biotechnology, Yeungnam University, Gyeongsan 38541, Republic of Korea; rakesharya101@yu.ac.kr; 2 Department of Biochemistry, All India Institute of Medical Sciences, New Delhi 110029, India; bimaljit2019@gmail.com; 3 Department of Orthopaedic Surgery, The Johns Hopkins University School of Medicine, Baltimore, MD 21205, USA

**Keywords:** tuberculosis, biofluids, exosomes, biomarkers, diagnostics, therapeutics

## Abstract

Tuberculosis (TB) is a major cause of morbidity and mortality and remains an important public health issue in developing countries worldwide. The existing methods and techniques available for the diagnosis of TB are based on combinations of laboratory (chemical and biological), radiological, and clinical tests. These methods are sophisticated and laborious and have limitations in terms of sensitivity, specificity, and accuracy. Clinical settings need improved diagnostic biomarkers to accurately detect biological changes due to pathogen invasion and pharmacological responses. Exosomes are membrane-bound vesicles and mediators of intercellular signaling processes that play a significant role in the pathogenesis of various diseases, such as tuberculosis, and can act as promising biomarkers for the monitoring of TB infection. Compared to conventional biomarkers, exosome-derived biomarkers are advantageous because they are easier to detect in different biofluids, are more sensitive and specific, and may be useful in tracking patients’ reactions to therapy. This review provides insights into the types of biomarkers, methods of exosome isolation, and roles of the cargo (proteins) present in exosomes isolated from patients through omics studies, such as proteomics. These findings will aid in developing new prognostic and diagnostic biomarkers and could lead to the identification of new therapeutic targets in the clinical setting.

## 1. Introduction

Tuberculosis, caused by *Mycobacterium tuberculosis* (*Mtb*), is still a serious health issue worldwide, responsible for ~1.3 million deaths in 2022 (95% uncertainty interval [UI]: 1.18–1.43 million). Globally, an estimated 10.6 million people (95% UI: 9.9–11.4 million) developed TB in 2022. Despite the development of effective TB medications and vaccinations, there were approximately 1.13 million deaths among HIV-negative individuals (95% [UI]: 1.02–1.26 million) and 1.30 million deaths (95% UI: 1.18–1.43 million) among HIV-positive individuals [1]. The use of rapid tests is growing, although it remains far too limited. A WHO-recommended rapid diagnostic test (WRD) was used as the initial test for 47% (3.5 million) of the 7.5 million people newly diagnosed with TB in 2022, up from 38% (2.5/6.4 million) in 2021 and 33% (1.9/5.8 million) in 2020. The coverage will need to more than double to reach the new target set at the 2023 UN high-level meeting on TB, which is 100% by 2027 [1].

A strategy for TB control for the early and precise diagnosis of active TB is essential in initiating prompt and effective treatment, which can stop the spread of the disease, cure it, and enhance patient outcomes. The other strategy is to prevent latent tuberculosis infection (LTBI) [2]. Various diagnostic methods rely on the detection of biomolecules perturbed during pathogen infections such as tuberculosis. Biomarkers are biomolecules (proteins, DNA/RNA, miRNA, cellular-based, etc.) that can be used for the monitoring of tuberculosis infection. Exosomal-based biomarkers for various infectious diseases, including tuberculosis, are gaining interest in the field of medical microbiology, using patient samples for diagnostic purposes.

### 1.1. Biomarkers

According to the National Institutes of Health (NIH), biomarkers are indicators of normal biological or pathological changes or pharmacological reactions to a treatment intervention that are generally measured and assessed. The Food and Drug Administration (FDA) defines a biomarker as an assessable indicator that has the potential to be useful throughout disease progression, the research and development of therapies, disease prognosis, diagnosis and monitoring, disease development, or the response to treatment. Biomarkers are categorized based on their clinical applications and characteristics, such as molecular, cellular, and imaging [3]. The major characteristics of an ideal biomarker include clinical relevance, high sensitivity and specificity, reliability, noninvasiveness, ease of modification, and cost-effectiveness [3]. Information about these biomarkers can be found in the MarkerDB database, which is a freely accessible electronic database containing consolidated information on all identified clinical biomarkers and a selected set of preclinical biomarkers. The database contains 26,374 genetic biomarkers and 154 karyotype biomarkers [4].

There are two major types of biomarkers: pathogen-generated and others produced by host cells upon pathogen invasion. The former involves products such as virulence factors released by *Mtb* during the infection of the host cell, which can be used as biomarkers [5]. These products are very specific to particular *Mtb* species and can be identified and quantified using a multiplex assay, which needs to be developed. During *Mtb* infection, both DNA and lipoarabinomannan (LAM) can be detected in urine [6,7]. The major drawbacks of these pathogen-generated biomarkers include variations in different populations and altered responses due to drug therapy. The latter category of biomarkers involves the INF-γ concentration, as quantified by the interferon-gamma release assay (IGRA) method; immunological markers, which include cytokines, e.g., IL-6 and tumor necrosis factor (TNF); the generation of antibodies against dominant *Mtb* antigens, e.g., MPT51 and malate synthase; and the protein profiling of host cells [8,9]. Thus, it is possible to develop a fingerprint of a series of molecules that are specific to particular stages of infection, e.g., latent vs. active TB.

There are different conventional diagnostic methods for TB, such as sputum acid fast bacilli (AFB) smear microscopy, Löwenstein–Jensen (LJ) culture, the tuberculin skin test (TST), IGRA, and polymerase chain reaction (PCR), which have limitations in sensitivity, specificity, and speed, especially in patients with extrapulmonary or paucibacillary TB [10]. Nucleic acid amplification tests, such as the GeneXpert *MTB*/RIF^®^ assay (Cepheid, Sunnyvale, CA, USA), are recommended by the World Health Organization (WHO) for the rapid diagnosis of TB and resistance to rifampicin, especially in regions with limited resources. Other tests include *Mtb* antigen-based skin tests (TBSTs); rapid diagnostic tests (RDTs), such as the TrueNat™ *MTB* Plus Assay (Molbio Diagnostics Private Limited, Goa, India); *MTB*-RIF Dx (for the detection of rifampin resistance); and TB blood tests (IGRAs), which are also recommended by the WHO. The lateral flow urine lipoarabinomannan assay (LF-LAM) has sensitivity of around 40% in detecting tuberculosis. As the test does not require sputum collection, LF-LAM may be the only way to diagnose tuberculosis in about 25 min when sputum cannot be produced [11]. During the last decade, several advancements in the fields of genomics and proteomics have been achieved to aid in our understanding of host–pathogen interactions. These methods are time-consuming, less sensitive, costly, and prevent TB from being diagnosed quickly. Therefore, identifying diagnostic markers (e.g., exosomal-based markers) for the quick detection of TB is extremely important.

### 1.2. Functions of Exosomal Cargo

The basis of infection by *Mtb* is the release of bacterial membrane vesicles (MVs) for the transmission of signals to nearby cells. These vesicles carry and transfer virulence factors, moderate bacterial binding and invasion inside the cell, cause cytotoxicity, and regulate the host immune response [12,13]. However, the biochemical, immunological, and genetic methods used for the detection and identification of bacterial products in biofluids are not always accurate; therefore, there is a need for the identification of intracellular pathogens or host-related deregulated molecules in secretory vesicles. This can be achieved by exosomes that are secreted by host cells, such as macrophages, which contain perturbed cargo due to infection and play a pertinent role in host–pathogen interactions [14,15]. Exosomes released by macrophages infected with *Mtb* or *Mycobacterium bovis* contain pathogen-derived antigens, and, as a result, these microvesicles can trigger innate as well as acquired immunological responses [16,17,18] and induce naïve cells to release proinflammatory cytokines. Exosomes containing mycobacterial contents have been identified in the serum of patients with active and latent TB infections (LTBIs) and reveal complex biomarker patterns across a spectrum of TB disease states [19].

Extracellular vesicles (EVs) are membrane-bound structures 30–5000 nm in diameter that are present in prokaryotes and eukaryotes. These vesicles are subcategorized into microvesicles, ectosomes, shedding vesicles, or microparticles, among others, based on their shape, size, morphology, origin, and mode of secretion [20,21,22,23]. Exosomes are small, single-membrane, secreted organelles of ~30 to ~200 nm in diameter that have the same topology as the cell from which they are derived [24]. They are enriched in selected proteins, lipids, nucleic acids (DNA/RNAs), and glycoconjugates; are secreted by almost all cell types; and are responsible for the regulation of many biological processes [24]. *Mtb*-derived exosomes are implicated in TB pathogenesis by delivering mycobacterial components to recipient cells [25]. Exosomes released from *Mtb*-infected macrophages contain *Mtb* components such as the 19-kDa glycolipid lipoarabinomannan (LAM), which inactivates macrophages and scavenges oxidative radicals, and *Mtb* proteins in exosomes from the serum of TB patients; these include the antigens 85b, Mycobacterial Protein Tuberculosis 64 (MPT64), GlcB, and BfrB, which hinder protective immune responses, affecting cellular immunity [18,26,27]. In addition, exosomes from TB patients also consist of sphingomyelins, phosphatidylcholines, phosphatidylinositols, free fatty acids, and triacylglycerols [28].

Exosomes are also important in cell–cell communication through the receptor-mediated transfer of proteins, lipids, and other genetic materials, e.g., between tumor and nontumor cells within the microenvironment [29]. They are involved in signaling and transferring cargo, influencing the immune response, extracellular matrix degradation, coagulation, cardiovascular function, resistance to drugs, and stem cell renewal [30]. They are present in most biofluids, such as serum, urine, cell culture supernatants, breast milk, ascitic fluid, bronchoalveolar lavage (BAL) fluid, amniotic fluid, malignant pleural effusions, semen, saliva, and synovial fluid [31]. On the other hand, ectosomes are vesicles of various sizes (0.1–1 μm in diameter) that bud directly from the plasma membrane and are shed into the extracellular space [32]. In contrast to living cells, ectosomes contain the phospholipid phosphatidylserine on their surface. These vesicles are long, considered artifacts, and confused with exosomes [32]. These vesicles are discharged upon the exocytosis of multivesicular bodies and with the cytoplasmic particles generated during apoptosis [32]. The largest EVs are apoptotic bodies (1–5 μm in diameter), which are formed during apoptosis and contain cellular contents enclosed by a membrane [33].

In the last two decades, exosomes have attracted attention for use as research tools; they are released by almost all cells present in the body and can act as potential sources of TB biomarkers [18]. Exosomes are rich in various types of biomolecules, such as nucleic acids (DNA, mRNA, and miRNA), proteins, lipids, enzymes, and metabolites, which reflect the physiological and pathological states of the cells from which they originate and can be easily identified [34]. The biomolecules isolated from exosomes have several advantages as TB biomarkers. First, exosomes are present in various biofluids and can be collected using various noninvasive methods. Second, they are stable and can withstand various conditions, including freezing and thawing cycles and storage at room temperature, making them more useful than other biomolecules [35]. Third, the exosomal cargo can be quantified using different techniques, such as Western blotting, enzyme-linked immunosorbent assays (ELISA), and mass spectrometry, depending on the abundance and specificity of the targets. Fourth, recent investigations have shown that exosomes also transport mycobacterial proteins [19,36]. Additionally, these methods do not carry a high risk of contamination compared to sputum microscopy and culture. Finally, the exosome content varies depending on the patient’s health status and the cell of origin [37].

The composition of the exosomal cargo varies greatly with the cell and tissue from which the cargo originates, and most of the cargo contains an evolutionarily conserved set of proteins. A single exosome has been shown to contain more than 20,000 proteins based on the size of the protein, its configuration, and its packaging parameters [38]. In a different study, another group described the changes in cellular and exosomal mRNA and miRNA content, as well as the functionality of the exosomal mRNA cargo [39]. Exosomes also contain double-stranded DNA (dsDNA) [40]. Exosomes transfer biomolecules from one cell to another through the trafficking of membrane vesicles, thereby inducing immune cells such as B cells and dendritic cells, and may play a significant role in modulating adaptive immune responses against pathogens [41]. Exosomes and other microvesicles also help cells to transfer less necessary or potentially harmful molecules, such as drugs, in neoplasia, and the export of chemotherapeutic drugs may facilitate cellular chemoresistance [42].

Different analyses of exoproteomes, such as nanoparticle tracking analysis (NTA), transmission electron microscopy (TEM), and ultracentrifugation, have shown that the exosomes secreted by mammalian cells share the most common features, such as shape, size, density, and total protein composition. Almost all exosomes have certain proteins on their surfaces that can serve as exosomal markers, and other proteins are found in the lumen. Importantly, these include cytoplasmic proteins (actin-binding proteins, tubulin, actin, Rab, and annexin proteins); proteins involved in signal transduction (protein kinases and heterotrimeric G-proteins); and heat-shock proteins (Hsp70 and Hsp90) [43]. MHC class-I molecules are found in the majority of exosomes [44]. Among the proteins in the Tetraspanin family, CD9, CD63, CD81, and CD82 are enriched in the membranes of exosomes and are mostly used as TB exosome biomarkers; these proteins are involved in immune cell signaling and modulation [45]. Many other exosomal proteins may represent the proteome of the originating cells from which they were derived; for example, the analysis of vesicles isolated from urine revealed a connection between exosomes containing aquaporin-2 (AQP-2) and the urogenital tract from which they originated [46]. Urinary vesicles were examined for their potential use in the detection of proteins from normal healthy subject samples, and the results indicated that exosomes might provide new biomarkers for kidney diseases [47]. As exosomes are also detected in the ascites fluid and serum of tumor patients, these biofluids can be used for diagnosis and biomarker analysis. Multidimensional protein identification technology (MudPIT) has been used to characterize proteins, which has resulted in the identification of more than 3000 exosomal proteins [48]. Moreover, the analysis of these exosomal biomolecules (proteins, metabolites, and nucleic acids), which also contain mycobacterial antigens that can modulate the immune system and can help to predict patient outcomes, such as the response to treatment or disease relapse, has been performed. Therefore, further research is warranted to develop noninvasive, cost-effective, and less time-consuming diagnostic tests based on exosomal proteins for the early detection and monitoring of TB.

## 2. Classification of Biomarkers

**Figure 1:**
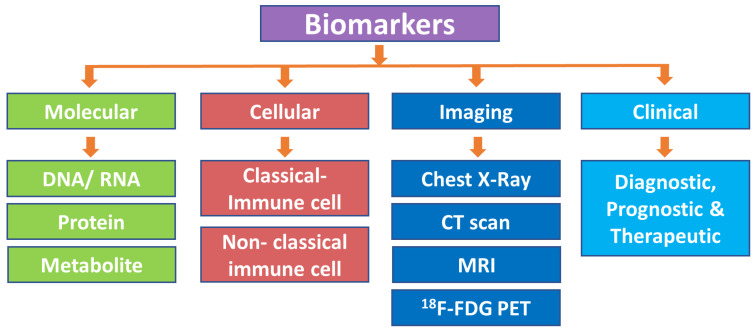
Classification of biomarkers. CT scan, computed tomography scan; MRI, magnetic resonance imaging; ^18^F-FDG PET/CT, ^18^F-fluorodeoxyglucose positron emission tomography/computed tomography.

Biomarkers have been categorized using various parameters based on their properties, molecular biology methods, and genetic and clinical applications [4] (Figure 1).

### 2.1. Molecular Biomarkers

#### 2.1.1. DNA/RNA-Based Biomarkers

DNA-based biomarkers are indicators of the biological state, whether normal or abnormal, and hold significant promise as diagnostic and prognostic tools for various diseases, including tuberculosis, cancer, cardiovascular disease, and neurodegenerative disorders. DNA-based biomarkers monitor genetic variations such as DNA mutations, single-polynucleotide polymorphisms (SNPs), and karyotyping. Recent advances in genetic technologies and nucleic acid amplification-based tests (NATs) for the detection of specific genomic regions of *Mtb*, such as conventional PCR, loop-mediated isothermal amplification (LAMP), GeneXpert *MTB*/RIF, and quantitative real-time PCR (qPCR), have been developed [49,50,51]. One of the most studied DNA biomarkers is IS6110, which is an insertion element that is found exclusively within members of the *Mtb* complex (MTBC) and has become a significant diagnostic tool in the identification of MTBC species. The ddPCR platform targeting IS6110 was evaluated in parallel using total DNA and exosomal DNA (exoDNA). The clinical performance of the ddPCR method was assessed with 190 respiratory samples from patients with suspected pulmonary TB and it was found that the sensitivity and specificity were 61.5% (95% CI 44.6–76.6%) and 98.0% (95% CI 94.3–99.6%) using total DNA, and 76.9% (95% CI 60.7–88.9%) and 98.0% (95% CI 94.3–99.6%) using exoDNA, when the results of droplet digital PCR (ddPCR) were compared with those of a mycobacterial culture [52].

Extracellular RNAs (exRNAs) are special types of RNA (e.g., miRNAs and lncRNAs) that are found within various tissues and biofluids, such as blood, saliva, urine, breast milk, and semen. These RNAs are carried in extracellular vesicles, exosomes, lipoproteins, and protein complexes and are proposed to play important roles in different biological processes, including intracellular communication, cell regulation in tuberculosis, cancer, etc. [53]. During *Mtb* infection, lung macrophages can release exosomes into the extracellular space that contain specific miRNAs. According to a previous study, monocyte-derived macrophages (MDMs) infected with *M. bovis* Bacillus Calmette-Guérin (BCG) secreted specific exosomal miRNAs, such as *miR-1224*, *miR-1293*, *miR-425*, *miR-4467*, *miR4732*, *miR-484*, *miR-5094*, *miR-6848*, *miR-6849*, *miR-4488*, and *miR96* [54]. In another study, macrophages infected with *Mtb* were shown to inhibit the exosomal encapsulation of certain miRNAs, which appeared to influence target genes linked to the immune response for surveillance and inflammation [55]. Additionally, exosomes released from *Mtb*-infected macrophages include a collection of particular host miRNAs and mycobacterial RNAs, which both contribute to the *Mtb* infection process and act as diagnostic biomarkers of TB disease [55]. A similar study showed comparisons of serum exosomal miRNA profiles among patients with active TB (ATB) or latent TB infection (LTBI) and healthy subjects. They found the specific upregulation of five exosomal miRNAs in the active TB group (*hsa-miR-28-3p*, *hsa-miR-193b-5p*, *hsa-miR-1246*, *hsa-miR-2110*, and *hsa-miR-370-3p*) and four exosomal miRNAs in the LTBI group (*hsa-let7d-5p*, *hsa-let-7e-5p*, *hsa-miR-140-5p*, and *hsa-miR-450a-5p*) and revealed that such studies can aid in the development of potential molecular targets for the discovery and diagnosis of active and latent TB infection [56]. It has been found that *miR-484*, *miR-425,* and *miR-96* are significantly increased in the serum of TB patients, with AUC (ROC) values of 0.72, 0.66, and 0.62, respectively, which are correlated with the infection level of TB and defined based on the smear positivity grade [57].

#### 2.1.2. Protein-Based Biomarkers

**Figure 2:**
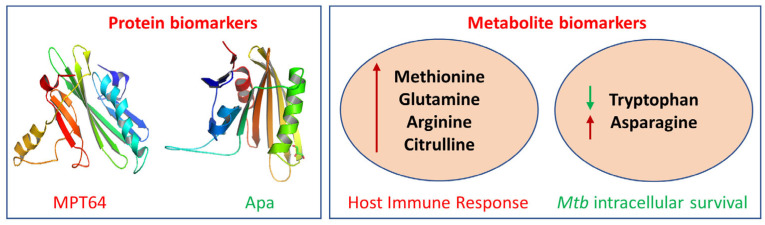
Examples of protein and metabolite biomarkers for tuberculosis: MPT64, Mycobacterial Protein Tuberculosis 64; Apa, Antigen 85 complex protein A; red arrow, upregulation; green arrow, downregulation.

Exosomes are enriched in proteins that are derived from their cells of origin, and the identification of deregulated proteins during infection reflects the disease state compared to healthy cell types [17]. Since *Mtb* causes intracellular infections, releasing mycobacterial components from the phagosome, most of the proteins related to mycobacteria are localized in exosomes [58]. Although the potential mechanism by which mycobacterial components are localized from multivesicular bodies (MVBs) into exosomes is not known, the isolation and enrichment of exosomes can lead to the identification of potential biomarkers for TB diagnosis. A study identified more than 250 potential biomarkers from exosomes derived and purified from *Mtb*-infected macrophages [59]. Moreover, biomarkers have been found in biofluids isolated from *Mtb*-infected animal models [60]. Since the abundance of these biomarkers is very low, by using high-throughput proteomic analysis tools with high sensitivity, it is possible to identify mycobacterial-specific protein biomarkers for TB diagnosis. The *Mtb* protein biomarkers MPT64 (Rv1980c, 24 kDa) serve as a pivotal component in immuno-chromatographic assays for the rapid identification of the MTBC [61] and alanine and proline-rich secreted protein (Apa, Rv1860, 45/47 kDa) functions in modulating the macrophage immune response, influencing proliferation and cytokine secretion [62] (Figure 2).

**Figure 3:**
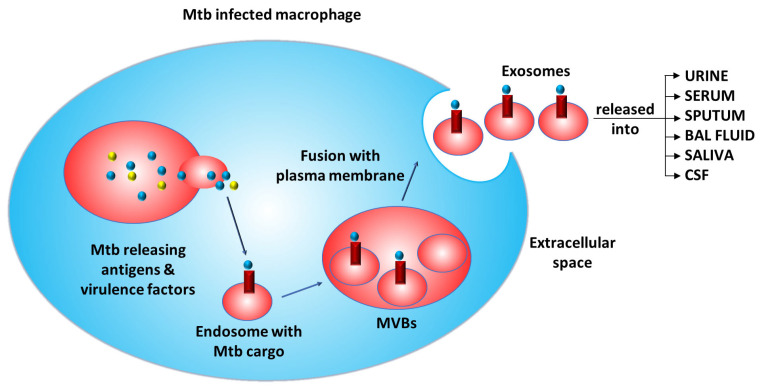
Mechanism of the release of exosomes containing cargo from *Mtb*-infected macrophages. (*Mtb*: *Mycobacterium tuberculosis*; MVBs: multivesicular bodies).

Additionally, exosomes encapsulate infectious proteins, RNA, virulence factors, and prions, which can be used for the development of tests against infectious agents. The problems of cost-effectiveness, sensitivity, time consumption, etc., posed by different methods can be overcome by using exosomes to detect active and latent TB infections. A study detected 33 unique *Mtb* proteins from human serum exosomes that can serve as potential biomarkers for active and latent TB [19]. In another study, the mycobacterial LAMS 19 kDa antigen (Rv3763) and the host exosomal marker LAMP-1 were detected via Western blotting [63]. Secreted proteins, including the Ag85 complex (Rv1886c, Rv0129c, Rv3804c), KatG (Rv1908c), CFP10 (Rv3874), and GroES (Rv3418c), were also detected in exosomes by Western blotting [60]. A recent study identified 40 *Mtb* peptides from 19 proteins using multiple reaction monitoring mass spectrometry (MRM-MS); these peptides most commonly copurified with the serum vesicles of patients with TB [36]. Thus, we can assume that the mycobacterial proteins secreted into the phagosome or cytoplasm are transported to endosomes via endocytosis and then form MVBs, fuse into intraluminal vesicles, and exit the infected cell via exosomes into different biofluids (Figure 3).

Exosomes and exoproteomes are being studied using techniques such as microscopy, dynamic light scattering (DLS), Western blotting, fluorescence-activated cell sorting (FACS), and mass spectrometry. Both the pathogen and host cell products are characterized as proteins, lipids [64], mRNAs, miRNAs [39], etc. The proteomic analysis of the exosomal cargo derived from urine and blood has been used to obtain biomarkers in various diseases, such as cancer, diabetes, and kidney diseases [65,66,67]. To examine the potential of exosomes derived from tumor cells, they were genetically modified to express a *Mtb* antigen, as a cancer vaccine aimed at overcoming the weak immunogenicity of tumor antigens [68]. In a different study, 287 vesicular proteins were identified with high confidence by four LC-MS/MS analyses. Furthermore, multiple vesicular proteins related to *Mtb* virulence have been discovered, which will aid in understanding the pathogenic mechanism of *Mtb* [69]. The main focus of future research is to characterize the exosomal cargo released during infection by *Mtb* to host cells, as this cargo is concentrated inside exosomes [60]. Several studies have shown that the exo-proteome is altered inside cells after infection with *Mtb*, and these altered cargos are subsequently released as exosomes [70]. A study revealed that U937 cells infected with *Mtb* were able to secrete abnormally large amounts of the Hsp16.3 protein in exosomes [71]. Therefore, a substantial number of Hsp16.3 proteins were detected in the blood exosomes of tuberculosis patients [71]. Furthermore, the identification and quantification of these biomarkers can be performed by high-throughput mass spectrometry-based technologies (Table 1).

#### 2.1.3. Metabolite-Based Biomarkers

Metabolites are small molecules that are byproducts of various biochemical processes in the body. In various phases of infection, the dynamics of the metabolite products produced by the interaction of *Mtb* with the host play a significant role in the stimulation and regulation of the host’s defense system. Immune cells also modify the cellular metabolism to create enough energy for host immune processes and to adapt defenses against infectious cells [83]. Metabolites can serve as valuable indicators of a person’s overall health and can be used to diagnose a disease, assess the response to treatment, and monitor disease progression for a wide range of diseases, including tuberculosis, cancer, diabetes, and Alzheimer’s disease. Various metabolites that have been identified in different studies have shown deregulation during tuberculosis infection [84]. One such category includes amino acids, which play an important role in tuberculosis biology, as they stimulate the host immune response (methionine, glutamine, arginine, and citrulline) and help *Mtb* to survive cellular stress (tryptophan and asparagine) [85] (Figure 2).

Methionine is an essential amino acid that plays an important role during infection [86]. After infection, the body produces more DNA, proteins, and other biomolecules to help in cell multiplication and T-cell proliferation and differentiation [86]. This can cause the body to experience an overload of energy and resources, leading to problems with metabolism. One important way that the body deals with this is by importing methionine and upregulating Slc7a5, which helps in the complete activation of T cells. Serum biomarkers such as methionine provide efficient antioxidant defenses by reacting with reactive oxygen species (ROS) and are potentially useful for adjunctive, rapid, and noninvasive pulmonary TB diagnosis [87]. Moreover, glutamine is important for the host’s defense against infection and helps to generate ATP through glutaminolysis, which then increases the levels of T-cell-derived cytokines such as IL-22, IL-17, and IFN-γ [88]. These cytokines play an important role in immunity against *Mtb* and may influence risk factors [89]. Furthermore, arginine metabolism occurs in the body when an individual is infected with *Mtb*. Macrophages release inducible nitric oxide synthase (iNOS) to generate large quantities of nitric oxide (NO), which aids in fighting infection [90]. When L-arginine was given as a supplement to TB patients, it was found to increase NO synthesis, which in turn helped to alleviate coughs and chest pain and eventually led to the clearance of sputum [91].

Another amino acid, citrulline, can be obtained from the diet or the conversion of ornithine or arginine using ornithine carbamoyl transferase or NO synthase, respectively [92]. Citrulline has shown strong antimicrobial activity by transferring nitrogen to mouse macrophages and T cells when there is a shortage of arginine. Citrulline has been shown to have antimicrobial activity through the use of arginine present in the cell and plays an important role in preventing *Mtb* infection [93]. Lower levels of citrulline might be harmful to patients with active tuberculosis [94]. Moreover, tryptophan has dual effects on tuberculosis infection, including being favorable for *Mtb* infection and the host response. Studies have shown that the serum tryptophan concentration is lower in TB patients than in LTBI patients [95]. However, kynurenine, a product of tryptophan, was expressed at higher levels in the serum of TB patients than in that of patients with latent TB [83]. Lower levels of tryptophan induce low levels of the enzyme indoleamine 2,3-dioxygenase 1 (IDO1), which in turn induces fewer *Mtb*-specific T cells. *Mtb* also produce tryptophan and convert it to kynurenine by IDO through a pathway similar to that used to fight the host immune response [96]. Since asparagine is not directly involved in the intracellular survival of *Mtb*, it helps in generating nitrogen sources such as ammonia to maintain the environmental pH [97]. *Mtb* assimilates asparagine and converts it to ammonia and aspartate, helping *Mtb* to survive acidic stress inside macrophages [98].

### 2.2. Cell-Based Biomarkers

#### 2.2.1. Classical Immune Cell-Based Biomarkers

Immune cell biomarkers have shown potential as diagnostic and prognostic tools for tuberculosis (TB). During exposure to *Mtb*, several immunological changes occur, such as the differentiation of CD4+ and CD8+ T cells from naïve T cells to terminally differentiated cells [99]. Therefore, these changes could be useful in characterizing the association and severity of different stages of tuberculosis. One such example is cluster of differentiation 69 (CD69), which is a costimulatory receptor and early marker of activation, and its increased levels are related to an increase in TB infection [100]. A study reported that the costimulatory molecule CD137, which helps in the activation, proliferation, and survival of T cells, is associated with tuberculosis infection in TB patients [101]. In another study, it was found that phenotypic alterations in *Mtb*-specific T cells were potential surrogate biomarkers for tuberculosis treatment efficacy and could help to distinguish between active TB (profiles: CD38^pos^, CD27^low^), cured TB (CD38^neg^, CD27^low^), and latent *Mtb* infection (CD38^neg^, CD27^high^) [102]. Similarly, another study showed that a member of the TNF-α superfamily, CD27, was able to distinguish between active TB patients and latent TB patients. Other studies have shown that the CD4 + CD27+ T-cell levels are greater in TB patients than in BCG-vaccinated individuals, but LTBIs exhibit intermediate CD27+ T-cell counts [103]. Another study showed that LTBI patients but not healthy or BCG-vaccinated individuals after TB treatment presented a CD4 cell subset, which was CD27-PC-1+, and demonstrated that *Mtb* antigens caused in vivo cell differentiation, which raises the possibility of these membrane markers being used to distinguish between people who have LTBI and healthy people, as well as to track the effectiveness of TB medication [104]. A recent study reported that TNF-α secretion from CD38^+^CD27^−^CD4^+^ T cells stimulated with ESAT6/CFP10 peptides had the best diagnostic accuracy, with a cutoff of 9.91% (exploratory: 96.67% specificity, 88.46% sensitivity; validation: 96.15% specificity, 90.16% sensitivity), and could discriminate treatment-naïve TB patients from individuals with treated TB after the completion of anti-TB treatment; moreover, validation was performed using whole blood in a blinded validation cohort comprising 165 individuals [105]. In another study, the IP-10 + IL-7 and/or IP-10 + BCA-1 marker combinations were proposed for use in serum samples to distinguish between active TB patients, latent TB patients, and healthy individuals [106].

#### 2.2.2. Nonclassical Immune Cell-Based Biomarkers

Nonclassical immune cells might offer *Mtb*-safe zones that are distant from the primary sites of the lesions, with less antigen presentation to elicit host immunological responses, which may encourage the *Mtb* to enter the dormant stage of latent tuberculosis [107]. Many biomarkers are secreted by various cell types during tuberculosis infection, such as epithelial cells, endothelial cells, fibroblasts, adipocytes, and glial and neuronal cells. A study revealed that the recruitment of polymorphonuclear leukocytes in response to *Mtb* infection depended on the production of the TLR2-dependent gene CXCL5 by lung epithelial cells [108]. When CXCL5 or its receptor CXCR2 was absent, mice exhibited improved pulmonary disease and increased longevity, indicating that *Mtb* may actively modulate immunological responses via epithelial cells to benefit them. Nitric oxide (NO), an important innate effector molecule that is needed to regulate *Mtb* replication, can be generated by epithelial cells [109]. While capable of directly killing *Mtb*, epithelial cells can work with lung macrophages to improve their antibacterial abilities in a way that is not dependent on NO [110]. Furthermore, in epithelial cells, lipocalin-2 is present, a protein that binds to *Mtb* proteins called mycobactins. These proteins tend to trap iron from the host, decreasing iron’s accessibility as a source of metabolism and preventing mycobacterial growth [111]. A particular increase in mycobacterial load was caused by lipocalin-2 deficiency in epithelial cells but not in pulmonary macrophages [112]. Recent research has demonstrated that after *Mtb* infection, local nonclassical *Mtb*-reactive CD8+ T lymphocytes efficiently recognize human lung epithelial cells and induce IFN-γ in a manner that is confined to the human leukocyte antigen [113].

Dendritic cell migration delays promote the growth of *Mtb* by decreasing Ag85B-specific CD4+ T-cell activation and proliferation [114]. Moreover, it was found that endothelial cells from healthy nontuberculous lung tissue contained *Mtb* DNA; therefore, endothelial cells may contribute to the persistence of this disease [115]. Fibroblasts infected with *Mtb* expressed less IFN-dependent MHC-II, which limited their ability to deliver antigens [116]. Further investigations demonstrated that CXCL8 limits the growth of intracellular *Mtb*, indicating that fibroblasts can modulate the immune response to TB by secreting CXCL8, which both induces chemotaxis and enhances the macrophage killing of *Mtb* [117]. Moreover, the development of pulmonary B-cell follicles is dependent on interleukin-23 and necessitates the fibroblast production of CXCL13. Interleukin-23 deficiency decreases the production of CXCL13, which in turn decreases the development of B-cell follicles and weakens the longstanding immunity against *Mtb* infection [118].

*Mtb* DNA was identified in non-TB patient adipose tissue from autopsy samples, providing the first evidence of *Mtb* persistence in adipocytes. In the same study, when both human adipocytes and 3T3-L1 murine adipose cell lines were infected with *Mtb* via the scavenger receptor, it was demonstrated that *Mtb* could remain dormant inside 3T3-L1 cells without replicating [119]. The ability of adipocytes to trigger immunological responses in response to both attenuated H37Ra infection and virulent H37Rv infection was further demonstrated by the discovery that they could synthesize NO and specific cytokines [120]. According to researchers, neurons can produce MHC-I and control immunological responses by engaging with CD8+ T cells directly when infected. Furthermore, the killing of intracellular bacteria by neurons is proposed to be IFN-γ-dependent. Thus, understanding how various nonclassical immune cells affect the immune system’s response to tuberculosis infection may help to identify new targets for anti-TB therapeutic strategies [121].

### 2.3. Imaging-Based Biomarkers

The diagnosis of TB is challenging since relevant and reliable data on TB diseases in human body fluids need to be obtained via clinical, instrumental, and radiological techniques. The different imaging biomarkers used include chest X-rays, CT scans, MRI, and ^18^F-FDG PET/CT. For example, a chest X-ray (CXR) is a rapid imaging tool used to detect lung abnormalities. They are the most commonly used imaging tests for TB diagnosis. The imaging biomarkers in chest X-rays include nodules, cavities, and infiltrates in the lungs. These findings can indicate active TB disease, although they may also be observed in other respiratory conditions. The CXR is an excellent screening tool for pulmonary tuberculosis due to its high sensitivity (87–98%, depending on how the CXR is interpreted) for TB diagnosis [122]. However, the accuracy of chest X-rays can be limited, especially in cases of extrapulmonary TB. Additionally, computed tomography (CT), is a medical imaging technique used to obtain detailed internal images of the lungs and can detect smaller abnormalities that may not be observed via chest X-rays. The imaging biomarkers in CT scans include a tree-in-bud appearance, nodules, cavities, and infiltrates. According to a previous study, high-resolution computed tomography (HRCT) had sensitivity and specificity of 90.9% and 96.4%, respectively, in identifying active PTB in smear-positive patients [123]. CT scans are particularly useful when chest X-rays are inconclusive or in monitoring the treatment response.

Magnetic resonance imaging (MRI) is not commonly used for TB diagnosis, but it can provide additional information in some cases. The imaging biomarkers in MRI include areas of inflammation and edema. One study revealed that MRI had sensitivity of 100% and specificity of 88.2% in detecting spinal TB [124]. MRI is particularly useful when TB affects the spine or central nervous system. Furthermore, ^18^F-fluorodeoxyglucose positron emission tomography/CT (^18^F-FDG PET/CT) is a noninvasive imaging technique that has been frequently utilized to distinguish between active and inactive PTB because active tuberculoma has a much greater standardized uptake value (SUVmax) than inactive tuberculoma. When an SUV_max_ of 1.05 (at 60 min) was used as the cutoff, the sensitivity and specificity were both 100% [125]. Imaging biomarkers play an important role in the diagnosis and management of TB by providing clinicians with valuable information about the location and extent of the infection, as well as the effectiveness of treatment. Chest X-rays, CT scans, MRI, and ^18^F-FDG PET/CT scans all have different advantages and limitations in the diagnosis of TB, and the choice of imaging modality depends on the clinical scenario. The use of imaging biomarkers, along with clinical data and data generated with analytical instrumentation, can improve the accuracy of TB diagnosis and the reliability of biomarker monitoring in TB patients.

### 2.4. Clinical Application-Based Prognostic, Diagnostic, and Therapeutic Biomarkers

A prognostic biomarker is a clinical or biological characteristic that provides information on likely patient health outcomes (e.g., disease recurrence) irrespective of the treatment. An increased serum Trp/Kyn ratio, a sign of elevated indoleamine 2,3-dioxygenase (IDO) activity, is associated with a poor prognosis in patients with tuberculosis. *Mtb* infection triggers the potent activation of IDO-1, thus elevating the kynurenine levels and impacting immune responses. Thus, the inhibition of IDO activity showed promise in TB management, as it reduced both clinical manifestations and the microbial burden [126]. It was previously reported that the activity of an immunoregulatory molecule, IDO, as measured by the ratio of kynurenine (Kyn) to tryptophan (Trp), was considerably greater in TB patients than in controls and was also higher in TB patients who died vs. TB survivors [127]. A different study revealed that 42 PTB patients had higher plasma chitinase enzymatic activity than did 30 healthy control subjects. Chitinase activity was shown to be positively associated with the radiographic TB severity and sputum smear positivity [128]. Diagnostic biomarkers are biological molecules that can indicate the presence of a disease. According to a previous study, a four-marker biosignature (for MMP-9, sIL6R, IFN-γ, and IL-2Ra) was able to detect TB in HIV-positive individuals, with an AUC of 0.96, sensitivity of 85.7% (95% confidence interval (CI) 42.1–99.6%), and specificity of 94.7% (95% CI 74.0–99.9%). However, in HIV-negative patients, the most promising two-marker biosignatures (sIL6R and sIL-2Ra) identified TB with an AUC of 0.76, sensitivity of 53.9% (95% CI 33.4–73.4%), and specificity of 79.6% (95% CI 70.3–87.1%) [129]. The severity of TB development may be determined by the presence of fibrinogen alpha chain (FGA) protein, which was reported to be more elevated in saliva and sputum than in serum in the TB group. FGA had an AUC of 0.765 and sensitivity and specificity for the detection of MDR-TB of 90% and 65%, respectively. PGLYRP2 (N-acetylmuramyl-L-alanine amidase), a peptidoglycan recognition protein, hydrolyzes tuberculosis peptidoglycan through its amidase activity, impacting cell wall integrity and potentially influencing bacterial growth and division. Compared to those in the drug-sensitive TB (DS-TB) group, the MDR-TB group had significantly greater PGLYRP2 levels, with sensitivity and specificity of 80%, and the AUC was 0.827 in differentiating between the MDR-TB group and the DS-TB group. Soluble CD14 (sCD14) is a putative activation marker of monocyte macrophages that plays a key role in monocyte activation. Monocytes move to the site of infection during the early stages of tuberculosis infection and develop into macrophages, which can trigger immunological responses and distinguish MDR-TB patients from healthy individuals with sensitivity and specificity of 85% and 50%, respectively, and an AUC of 0.655 [130].

The five identified TB-related proteins (alpha-1-antichymotrysin, plasminogen, macrophage-capping protein, f-actin-capping protein subunit beta, and profilin-1) identified by label-free liquid chromatography with tandem mass spectrometry, using saliva from 22 adults with symptoms of TB, with an AUC greater than 0.8, play a crucial role in enzyme regulation, immune system activation, and inflammation [131]. Therapeutic biomarkers such as proteins, miRNAs, and lipids are useful in the treatment of diseases. Moreover, these methods are crucial in assessing the clinical data that are used for targeted therapies [132]. Exosomes play crucial roles in tuberculosis (TB) by serving as carriers for TB-related molecules and affecting host cells. The main clinical applications of exosomes include biomarker studies, cell-free therapeutic agents, drug delivery mechanisms, exosome dynamics, and vaccine development.

## 3. Methods of Isolating Exosomes from Biofluids

Exosomes have emerged as good sources of biomarkers and novel therapeutic tools for different types of diseases. However, due to the limitations, such as their low abundance and heterogeneity in size, exosome isolation and their potential use are still challenging processes. Although ultracentrifugation is the gold-standard method for exosome isolation from cell culture supernatants, no standardized methods of obtaining biofluids are available. The nature of biofluids is complex and specific in terms of their composition and physical properties, which is a challenge in isolating pure exosomes. Additionally, some isolation methods affect downstream RNA or proteomic profiles and create a technical barrier to the generation of reproducible results [133].

The different types of biofluids, such as sputum, serum, urine, ascites, amniotic fluid, breast milk, bronchoalveolar lavage (BAL) fluid, and cerebrospinal fluid, can be collected from those with suspected TB by using invasive or noninvasive procedures. There are various methods available for the isolation of exosomes from these biofluids; these methods involve various sophisticated and/or easy steps, such as ultracentrifugation, size exclusion chromatography, polymer-based methods, ultrafiltration, commercial polymer-based precipitation, immunoaffinity capture, and microfluidics [134].

The gold-standard and most widely used exosome isolation method is ultracentrifugation (UC), which separates exosomes based on differences in size and density [135]. The procedure involves two major steps: first, cell debris, dead cells, large EVs, protein aggregates, and lipoproteins are removed at 300–400× *g* for 10 min, followed by 2000× *g* and 10,000× *g*; second, exosomes are separated at an ultrahigh speed of 100,000–200,000× *g* for 70–120 min; finally, the pellet is washed with PBS to obtain purified exosomes for further downstream processing, such as characterization and proteomic analysis. The exosomes are characterized based on their size (20–250 nm) and the presence of common surface protein markers such as Alix, TSG101, flotillin-1, CD9, CD63, and CD81. This method has several limitations, including its time consumption, cost, physical damage, contamination with lipoproteins, etc. [136]. To improve the purity of exosomes, ultracentrifugation combined with a sucrose density gradient can be used as an alternative method [137].

The size exclusion chromatography method makes use of the size differences between exosomes and other components in biofluids. The basic principle is that larger macromolecules, or EVs, are not able to enter the porous gel matrix or enter the mobile phase; rather, the exosomes remain in the gel and are ultimately eluted [138]. The advantage of using this method is that the integrity of the exosomes, such as their size and structure, is maintained, and the process is fast, easy, and inexpensive. The only disadvantage of this method is that they can be mixed with other impurities of similar sizes [139]. Currently, the commercially available SEC principle-based columns include qEV separation columns, EVSecond purification columns, and Exo-spin exosome purification columns [138].

In the other technique, exosomes are wrapped in an aqueous polyethylene glycol (PEG) solution, during which exosome aggregates are easily precipitated by centrifugation at 1500*× g*. The advantages include the simultaneous processing of multiple samples; being easy to use, faster, and cheaper; and the maintenance of exosome integrity. However, the purity and specificity are lost due to the coprecipitation of non-exosomal proteins, antibodies, and viral components [138]. Despite its high yield, this method results in low-quality exosome isolation, which are not suitable for further proteomic analysis. Nevertheless, this superhydrophilic polymer is efficient in clinical research settings, and combining this material with other techniques, such as immunoaffinity assays, makes it an attractive tool for crude and fast exosome extraction and analysis [140].

The ultrafiltration (UF) method depends on the use of membranes with various molecular weight cutoff (MWCO) values to separate exosomes of a specific diameter. Membrane filters with pore widths of 0.8 and 0.45 µm are used to remove larger particles first, producing a filtrate that is relatively rich in exosomes. Then, the smaller vesicles are removed from the filtrate by passing them into a waste eluate through membranes whose pores are smaller than those of the targeted exosomes (0.22 and 0.1 µm). The filtrate is then concentrated by repeatedly passing it through the exclusion filter and finally separated using a membrane with a diameter of 50–250 nm. Exosome recovery depends on the type of filter used, and the most effective recovery is achieved with cellulose membranes with a pore size of 10 kDa [141]. This method uses ultrafiltration tubes, which are inexpensive and highly efficient without affecting exosome activity. The disadvantages of this method include low purity (significantly contaminated by non-exosomal free-floating humoral peptides such as alpha-1-antitrypsin and albumin) and nonspecific interactions with ultrafiltration membranes, which decrease the recovery rate [141].

The immunoaffinity capture method uses surface protein markers, such as CD9, CD63, and CD81 (tetraspanin), which are specific to the capture of exosomes from biofluids, using antibodies against these proteins. Isolation can be achieved by incubating the sample with magnetic beads or gold-loaded ferric oxide nanocubes, which are coated with antibodies against surface proteins. This method can be employed to capture markers from parent cells, such as epithelial cellular adhesion molecule (EPCAM) [142], or exosome-binding molecules, such as heat-shock protein [143] and heparin [144]. By using quantitative detection and analysis, it is evident that, to obtain the same yield of exosomes via this method, a very low sample volume is needed compared to that needed for ultracentrifugation. For example, the amount of RNA recovered from 400 μL of plasma by this method is equal to the amount obtained by the ultracentrifugation of a 2.5 mL sample [145]. This method can be combined with UC to obtain exosomes of high purity. The disadvantages associated with these methods include the disintegration of the antibodies during storage and the fact that the selection of marker-specific antibodies may not reflect the complete picture of exosome biology or expense [146].

There are many commercial kits available that have advantages over all other methods, such as time savings, ease of use, high yields with a smaller sample volume, and better exosome integrity. Some examples are miRCURY, ExoQuick, the Invitrogen Total Exosome Isolation Reagent, the MagCapture™ Exosome Isolation Kit PS (Wako), Minute™ Hi-Efficiency, the Exosome Precipitation Reagent (Invent), and the exoEasy Maxi Kit (QIAGEN). Due to differences in isolation methodology, these kits carry certain disadvantages, such as high costs and low purity [147,148].

Microfluidics is a high-throughput technique in which microfluidic devices are used to isolate exosomes based on size, density, and immunoaffinity [148]. The most commonly used method is the immuno-microfluidic method, in which exosomes are separated by the specific binding of antibodies immobilized on chips to exosome surface-specific markers-for example, ExoChip [148] with a CD63 antibody. The advantages of this approach include efficient and fast processing, high purity, and the use of very low sample volumes (10 µL). The disadvantage of this method is the need for specialized equipment, which is expensive and complex [148].

## 4. Development of Biomarkers: Discovery and Validation Process

Mass spectrometry-based proteomics techniques have shown promise for the identification of new biomarkers, but they have not added much to the arsenal of diagnostic tools. Six crucial process components, viz. candidate identification, qualification, verification, research assay optimization, biomarker validation, and commercialization, can now be combined to create a complete biomarker pipeline. An improved experimental study design should increase the effectiveness of biomarker development, facilitate the delivery and deployment of novel clinical tests, and increase the understanding of the overall process of biomarker discovery and validation, as well as the difficulties and strategies inherent in each phase [149]. The primary tool for proteome discovery is mass spectrometry, which generates mass spectra that plot the mass-to-charge ratio of the detected ions, which can be analyzed using the isotope distribution, precise mass, amino acid sequence data, and tandem MS/MS. Proteins are enzymatically broken down into their constituent peptides to identify the components of these extremely complex mixtures. For unbiased proteomic biomarker identification, the MS/MS analysis of selected spots from differential proteins combines pattern-based and identity-based approaches [150].

**Figure 4:**
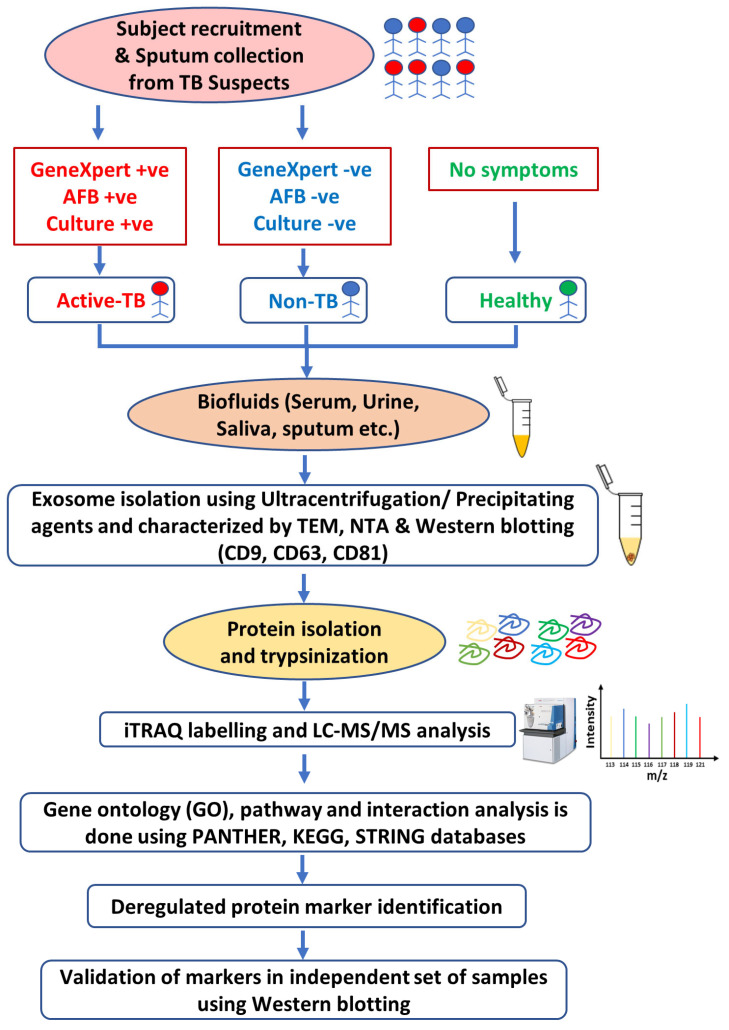
Workflow of discovery (iTRAQ) and validation (WB) of exosomal biomarkers from biofluids of tuberculosis patients.

The discovery and validation of protein biomarkers started with the isolation of exosomes from suspected TB and healthy subjects. Sputum samples are used for acid-fast bacilli (AFB) smear microscopy, LJ slant cultures, and GeneXpert to classify subjects as active TB or non-TB [151]. Exosomes are usually isolated using ultracentrifugation or by adding commercially available precipitating agents, such as ExoQuick^®^ (System Biosciences, Palo Alto, CA, USA). The isolated exosomes are characterized using different methods, such as transmission electron microscopy (TEM) to determine their size and integrity; nanoparticle tracking analysis (NTA) to analyze their population size; and Western blotting to detect specific surface marker proteins, such as CD9 and CD63 [151]. Proteins are usually isolated, denatured, reduced, alkylated, and trypsinized for further processing via iTRAQ labeling. iTRAQ utilizes isobaric reagents to label the primary amines of peptides and proteins. Peptides are fractionated using a strong cation exchange (SCX) column and analyzed via LC-MS/MS. The raw data are further analyzed to identify deregulated proteins associated with diseased conditions, such as tuberculosis. The area under the curve (AUC) of the receiver operating characteristic (ROC) curve can be calculated for the protein markers, and a value of 0.5–1 is considered to indicate statistical significance [152]. Gene Ontology (GO) enrichment, protein–protein interaction (PPI) networks, and pathway analyses can be performed to determine the involvement of these biomarkers in various biological processes or pathways related to different disease conditions. The protein biomarkers are ultimately validated in independent sets of samples, usually by Western blotting [151] (Figure 4).

Due to the increased interest in biomarkers, the development of new technologies and discovery methodologies has resulted in a significant increase in the number of proteins that have been identified as potential biomarkers for a variety of diseases. The crucial challenge in biomarker development has been finding the few candidates with performance traits that need comprehensive validation. Qualification is the process by which potential candidates identified using biological materials and discovery-oriented methods are converted into candidates utilizing verification-oriented methods and materials [149]. The test to be used for verification investigations, which will likely be different from the discovery assay, must demonstrate that the differential expression is still detectable for verification to be successful. Verification is a crucial step in the discovery process since it offers a more accurate quantification of candidate biomarkers than discovery usually does. It is conducted with samples that closely resemble the population that would be tested in a final clinical test, and it reintroduces the variation that was meticulously minimized during discovery and qualification [149]. To diagnose, stage, screen, predict, and track the progression of a disease, as well as to track therapy efficacy and patient adherence, biochemical markers are used [153]. By evaluating the test’s sensitivity, specificity, likelihood ratio, and receiver operating characteristic (ROC) curve, the diagnostic accuracy and predictability can be assessed [154].

## 5. Future Perspectives and Conclusions

In this review, we comprehensively summarize the recently revealed aspects of biomarkers and their history, classification, isolation, discovery, and validation processes. Biomarkers are excellent clinical tools that are relatively new and are used to diagnose, predict, and treat a variety of diseases, such as tuberculosis, cancer, and other diseases. The use of biomarkers in research on many aspects of illness, medication development, and the potential repercussions of treatment is unclear. A potential biomarker should be considered an ideal biomarker because of its clinical relevance, high sensitivity and specificity, reliability, noninvasiveness, ease of modification, cost-effectiveness, etc. This review focuses on exosomal biomarkers such as DNA/RNA, proteins, and metabolites that are altered during tuberculosis infection. However, most of the work performed on exosomes has involved release from in vitro cell lines. Meanwhile, the biological function of exosomes in complex cellular systems has yet to be explored. Many studies have demonstrated that exosomes are present in many biofluids under both healthy and diseased conditions. Moreover, the analysis of exosomes isolated from biofluids could reveal the source of biomarkers, since these vesicles reflect the molecular composition of the secreting cell from which they originated. In the urine samples of patients with tuberculosis, urogenital cancer, etc., the population of exosomes increases, which indicates a diseased condition. Additionally, the exosome cargo could be useful in differentiating between patients with diseased conditions (active TB), patients without TB (non-TB), and healthy individuals. The potential drawbacks or disadvantages associated with the use of exosomes in the diagnostic process that limit their clinical application include their storage stability, low yields, low purity, membrane integrity, aggregation, and the weak targeting of exosomes [138].

Moreover, for the analysis of exosomal proteins, several advancements have been made in two-dimensional gel electrophoresis (2DE), MALDI and SELDI, and nano-LC-MS/MS. Furthermore, nano-LC-MS/MS coupled with LTQ-Orbitrap has been used for shotgun proteomics, which can improve the speed, mass accuracy, and resolving power. This progress has resulted in the identification and characterization of a large number of proteins in exosomes isolated from different biofluids. Due to variations in populations, food habits, stress conditions, physiological conditions, environmental factors, and genetic backgrounds, there are differences in exoprotein profiles across individuals. A variational analysis is needed for future clinical applications. The present challenge for the use of biomarkers in clinical research is the development of accurate isolation techniques that are repeatable and detection methods that are compatible with current procedures. The development of techniques that can use readily accessible biofluids could lead to novel approaches to disease prognosis and diagnosis. Advances in proteomics and metabolomics could offer a means to find multiplex applications for disease diagnostics. Moreover, in many disease conditions, the exosomes released by infected host T cells contain pathogen-related biomolecules, which establish the basis for a regulatory role in the immune response against the pathogen. Exosomes regulate both the innate and adaptive immune responses against pathogens through various pathways. Therefore, exosomes may be an important key factor for the identification of new diagnostic and prognostic biomarkers and may also help in the development of vaccines.

## Figures and Tables

**Table 1:** *Mtb*-specific proteins identified in different studies.

SN	Total/Subproteome	Proteins	Method of Isolation	Instruments Used	Ref.
1	Sputum/Serum	Rv3804c-FbpA		Spectrophotometer	[72,73]
2	Sputum/Serum	Rv1860-Apa	Ag/Ab assay	Spectrophotometer	[74]
3	Serum	Rv0440-GroEL2		Spectrophotometer	[75]
4	CSF/Serum	Rv1837c-GlcB, Rv2031c-HspX, Rv0934-PstS1		Spectrophotometer	[76]
5	Serum	Rv2244-AcpM, Rv3804c-Ag85a, Rv1886c-Ag85b, Rv0129c-Ag85c, Rv1860-Apa, Rv3841-BfrB, Rv1827-Cfp17, Rv0350-DnaK, Rv0363c-Fba, Rv1837c-GlcB, Rv3418c-GroES, Rv2031c-HspX, Rv0066c-Icd2, Rv1908c-KatG, Rv1980c-Mpt64, Rv3248c-SahH, Rv0009-PpiA	ExoQuick	LTQ linear ion trap	[19]
6	Serum	Rv0129c-Ag85c, Rv1837c-GclB, Rv1860-MPT32, Rv1980c-MPT64, Rv2031c-HspX, Rv2376c-Cfp2, Rv3248c-SahH, Rv3418c-GroES, Rv3841-BfrB, Rv0350-DnaK, Rv1886c-Ag85B, Rv3874-Cfp10, Rv3875-EsxA, Rv2220-GlnA1, Rv3441c-MrsA, Rv0009-PpiA, Rv2244-AcpM, Rv3804c-Ag85A, Rv1827-GarA	ExoQuick	Xevo TQ-S mass spectrometer	[36]
7	Plasma	Hsp16.3	Ultracentrifugation		[71]
8	Urine	Rv1656-ArgF, Rv3341-MetA, Rv2392-cysH	Ultrafiltration	LCQ-DECA XP	[77,78]
9	Urine	Rv1681-MoeX		LCQ-DECA XP	[79]
10	Urine	Rv0014c-PknB, Rv2748c-FtsK, Rv1664-Pks9, Rv1161-NarG, Rv2490c-PE_PGRS43, Rv0578c-PE_PGRS7	Ultrafiltration	LTQ-Orbitrap Velos Pro	[80]
11	H37Rv-infected J774 cells and CFP-treated J774 cells	Rv0129c-Antigen 85C, Rv0211-PckA, Rv0350-DnaK, Rv0462-LpdC, Rv0896-GltA2, Rv0934-PstS1, Rv1448-Tal, Rv1827-Cfp17, Rv1837-GlcB, Rv1860-Apa, Rv1886c-Antigen 85B, Rv1908c-KatG, Rv1926c-Mpt63, Rv1932-Tpx, Rv1980c-Mpt64, Rv2031c-HspX, Rv2220-GlnA1, Rv2244-AcpM, Rv2376c-Cfp2, Rv2467-PepN, Rv2780-Ald, Rv2878c-Mpt53, Rv3248c-SahH, Rv3418 -GroES, Rv3804c-Antigen 85A	Filtration and ultracentrifugation	LCQ DECA XP	[60]
12	Culture	Rv2244-AcpM, Rv1860-Apa, Rv1793-FadA3, Rv0363c-Fba, Rv3804c-FbpA, Rv1908c-KatG, Rv2945c-LppX, Rv3763-LpqH, Rv0040c-Mtc28, Rv1017c-Prs, Rv0934-PstS1, Rv3846-SodB, Rv1793-EsxN, Rv2220-GlnA1	Density gradient ultracentrifugation	LTQ-Orbitrap Velos	[69]
13	Cerebrospinal fluid	Rv3875-ESAT6	1D-2D PAGE	Spectrophotometer	[81]
14	Cerebrospinal fluid	Rv2623-TB31.7		Spectrophotometer	[82]
